# P-60. Evaluation of clinical outcomes in Methicillin-Resistant Staphylococcus aureus bacteremia with earlier vs. later initiation of dual antimicrobial salvage therapy

**DOI:** 10.1093/ofid/ofaf695.289

**Published:** 2026-01-11

**Authors:** Victoria Sanderford, Sarah Alnami, Susan Spencer, Nikko Rowe A Tabliago, Rachel Burgoon, Taylor Morrisette, Richard R Lueking

**Affiliations:** Medical University of South Carolina, Charleston, SC; Medical University of South Carolina, Charleston, SC; MUSC, Charleston, South Carolina; Medical University Of South Carolina, Charleston, South Carolina; Medical University of South Carolina, Charleston, SC; Medical University of South Carolina College of Pharmacy, Charleston, South Carolina; Medical University of South Carolina, Charleston, SC

## Abstract

**Background:**

Treatment of Methicillin-Resistant Staphylococcus aureus (MRSA) bacteremia remains a persistent challenge in clinical practice, given its association with high morbidity/mortality. Despite antibiotic therapy guided by in vitro susceptibility, delayed culture clearance is common and may be linked to sub-optimal outcomes. Current guidelines recommend considering salvage therapy with ≥ 7 days of persistent bacteremia, but data on the impact of earlier initiation is limited.

**Figure:**
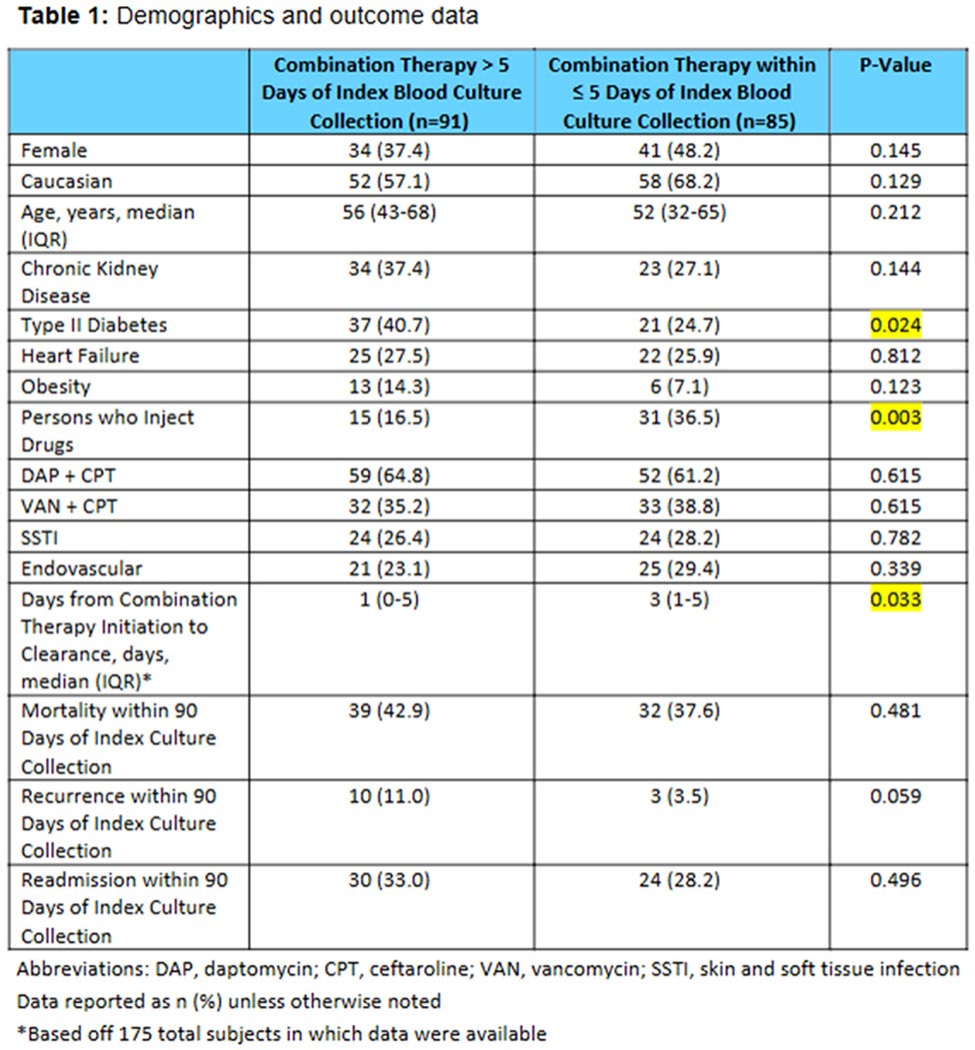

**Figure:**
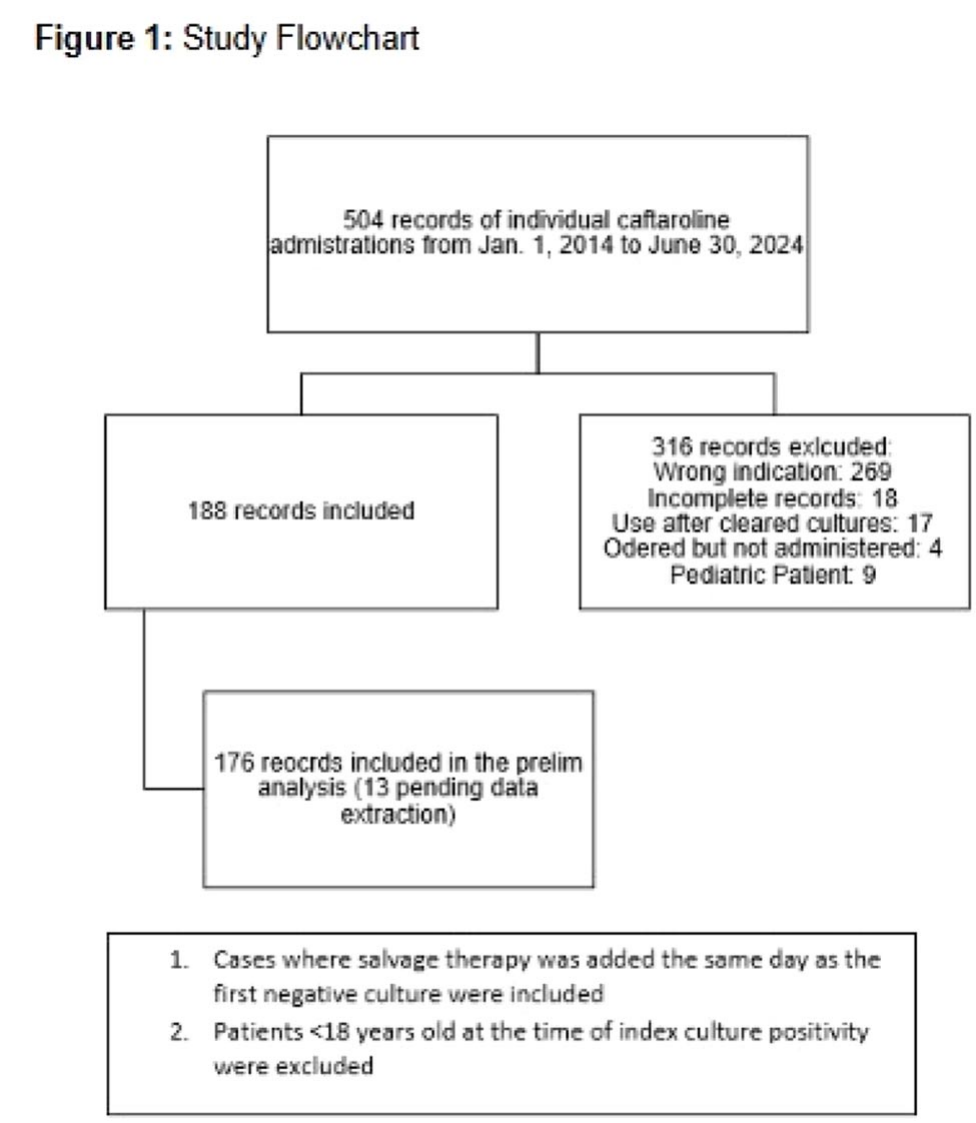

**Methods:**

We conducted a retrospective, observational cohort study of adult patients with MRSA bacteremia treated with ceftaroline-based combination salvage therapy at MUSC Health between January 1, 2014 and June 30, 2024. Patients were identified across 10 acute care hospitals using electronic health record query via SlicerDicer. Patients were excluded if ceftaroline was used for indications other than bacteremia, if dual therapy was initiated after culture clearance or for another indication, or if records were incomplete. Patients were stratified by timing of ceftaroline initiation: ≤5 days vs >5 days from the index positive culture. Primary endpoints included 90-day microbiologic recurrence, readmission, and all-cause mortality. Secondary outcomes included time from combination therapy initiation to culture clearance.

**Results:**

Among 504 patients who received ceftaroline during the study period, 188 met inclusion criteria. Preliminary analysis of 176 patients revealed no statistically significant difference between early (≤5 days) and late ( >5 days) initiation groups in terms of 90-day microbiologic recurrence (3.5% vs 11.0%, p=0.059), readmission (28.2% vs 33.0%, p=0.496), or all-cause 90-day mortality (37.6% vs 42.9%, p=0.481). Statistically significant differences were observed for median days to clearance (3 vs 1, p=0.033) and prevalence of persons who inject drugs (36.5% vs 16.5%, p=0.003).

**Conclusion:**

Preliminary data suggest that earlier initiation of ceftaroline-based salvage therapy (≤5 days) is not associated with statistically significant differences in 90-day microbiologic recurrence, readmission, or mortality, although numeric trends favor earlier initiation. Notably, earlier salvage therapy was more frequently employed in patients who inject drugs.

**Disclosures:**

Rachel Burgoon, Pharm.D., Merck: Grant/Research Support Taylor Morrisette, PharmD, MPH, AbbVie Inc: Advisor/Consultant|AbbVie Inc.: Grant/Research Support|Copeland, Stair Valz & Lovell: Expert Testimony|Infectious Diseases Special Edition: Honoraria|Stellus Rx: Grant/Research Support

